# Supplementary figures and images for: Environmental effects on the spread of the Neolithic crop package to South Asia
Source: PLoS One. 2022 Jul 11;17(7):e0268482. doi: 10.1371/journal.pone.0268482 (PMC9273075; doi:10.1371/journal.pone.0268482)

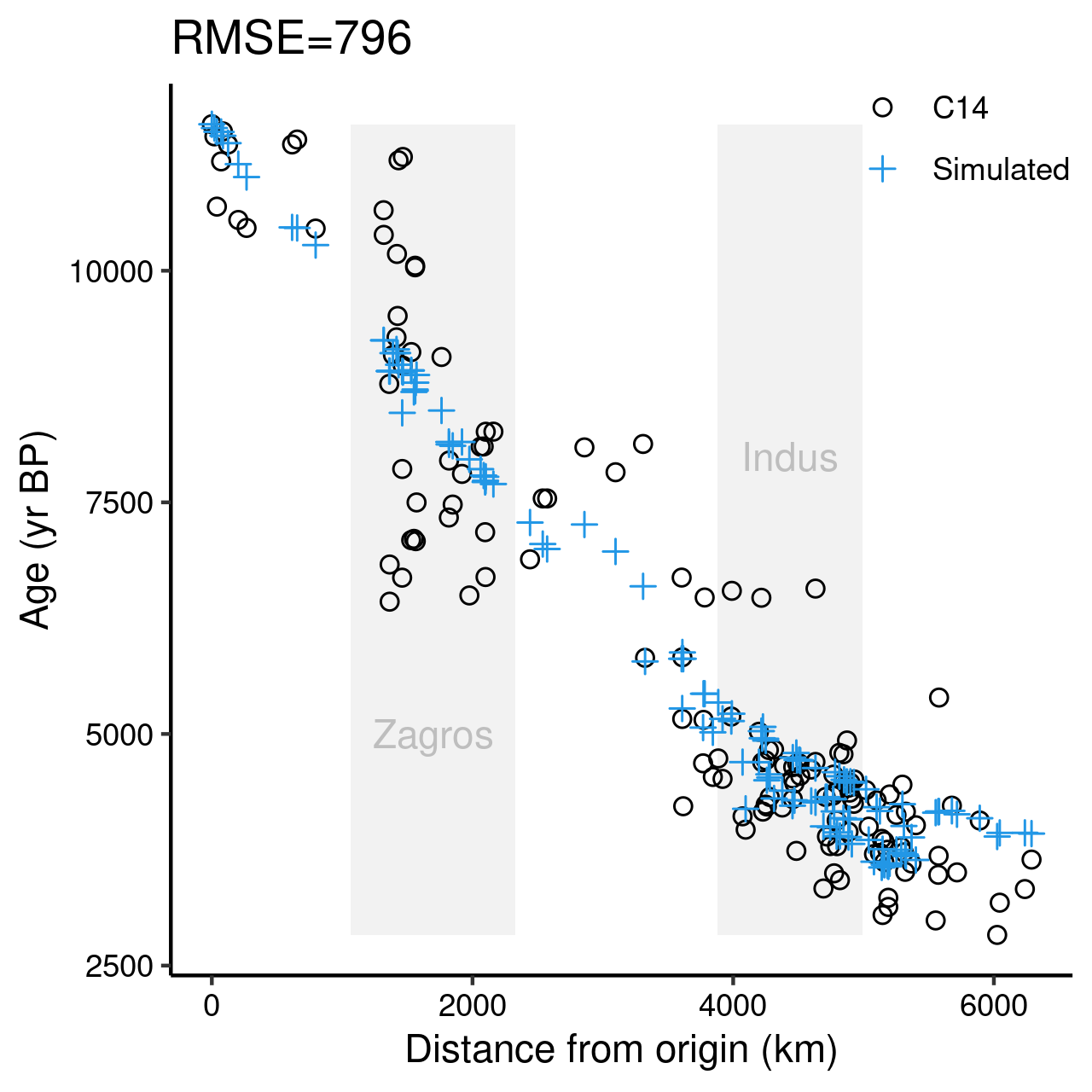

Supplement: S1 Fig — Comparison between the radiocarbon dates (median of the calibrated distribution) and the simulated arrival times using the optimal parameter set and the site of Dhra, Jordan, as the origin. The approximate distances to the Zagros Mountains and the Indus Valley are also shown. (TIF) [file pone.0268482.s001.tif]

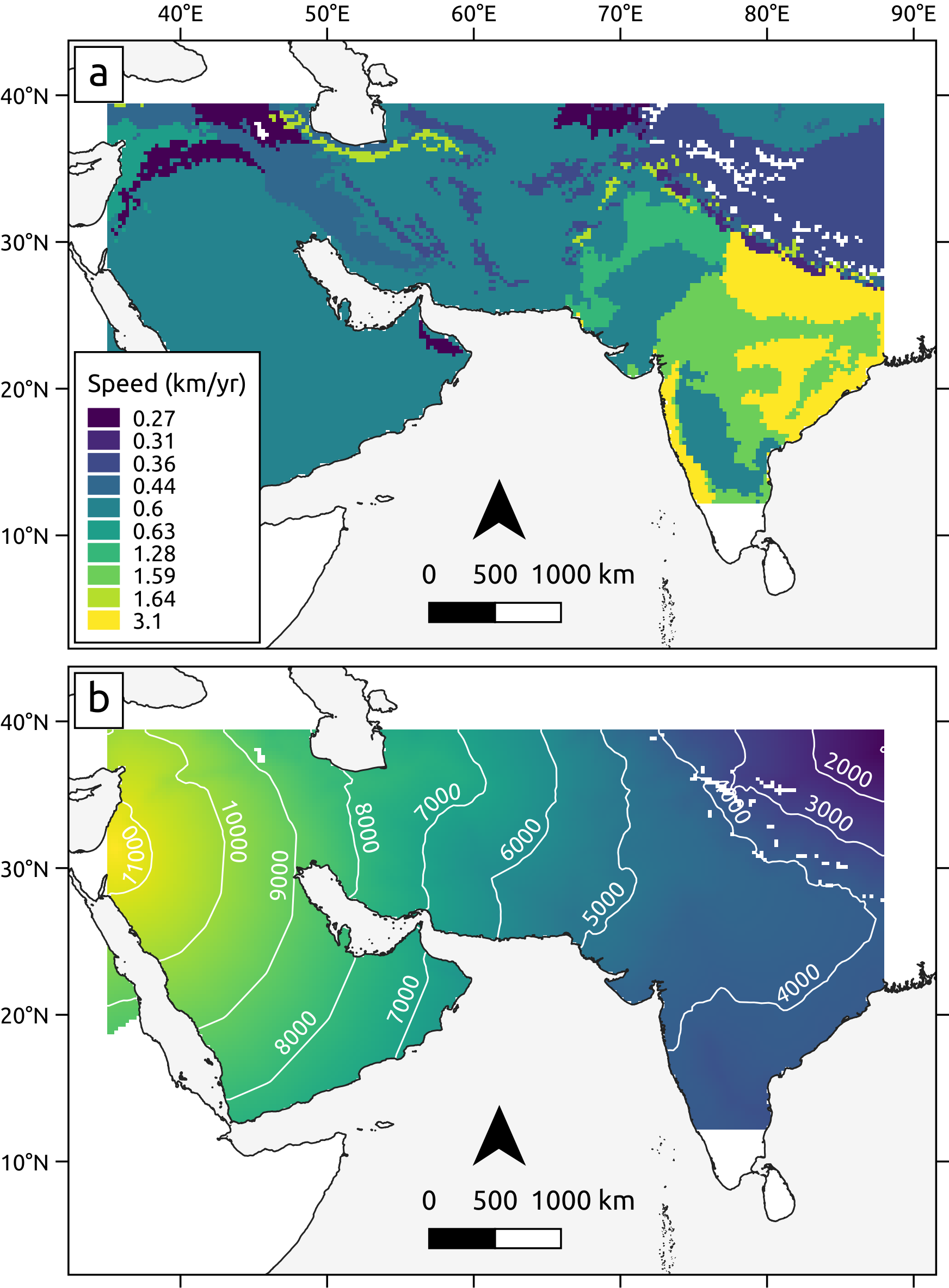

Supplement: S2 Fig — a) Simulated speeds of advance for each terrestrial ecoregion according to the optimal parameter set using the site of Dhra as the origin. b) Simulated arrival times from Dhra with contour lines (yr BP) shown every 1000 years. (TIF) [file pone.0268482.s002.tif]

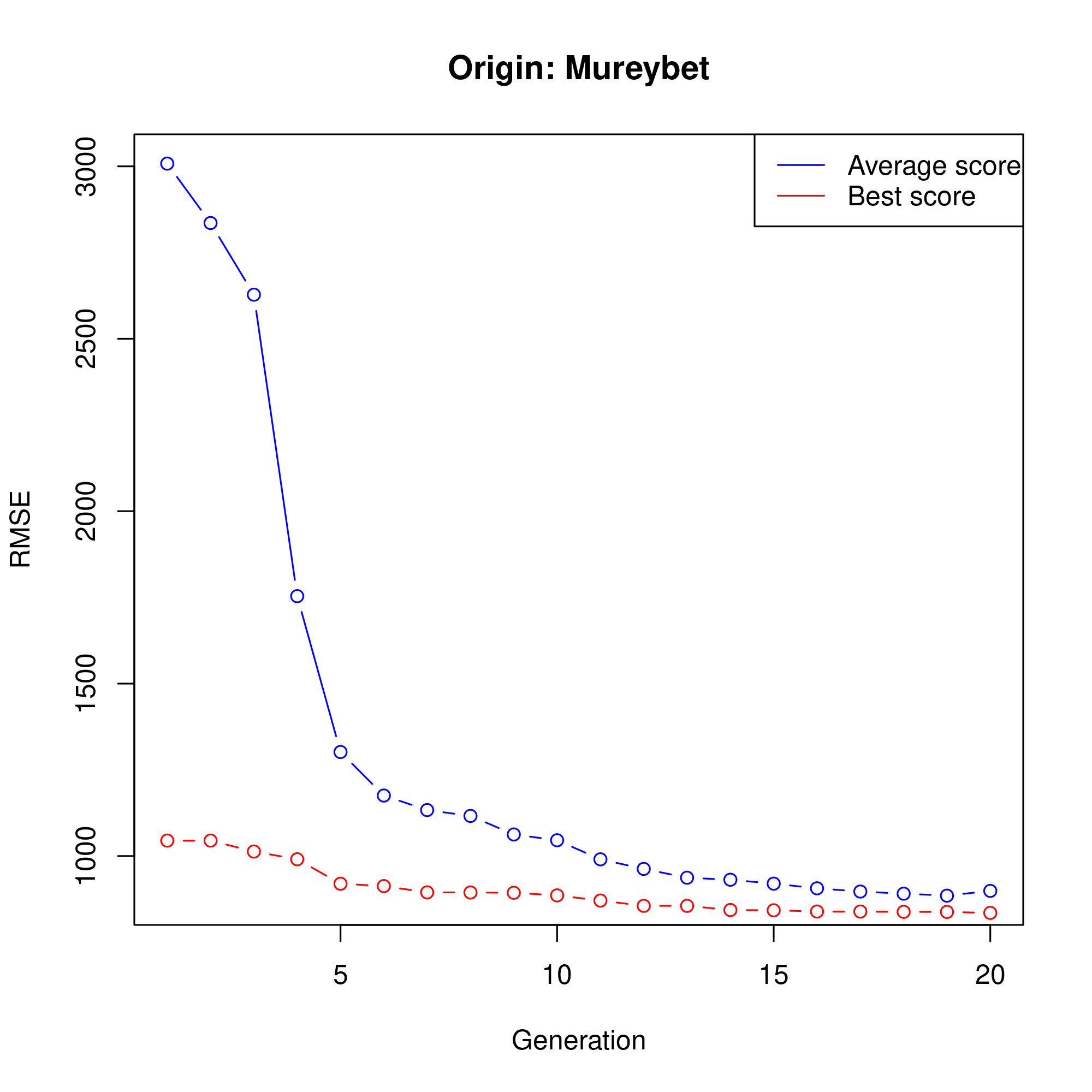

Supplement: S3 Fig — The average and the best (lowest) RMSE between simulated and radiocarbon dates are shown for each generation. (TIF) [file pone.0268482.s003.tif]

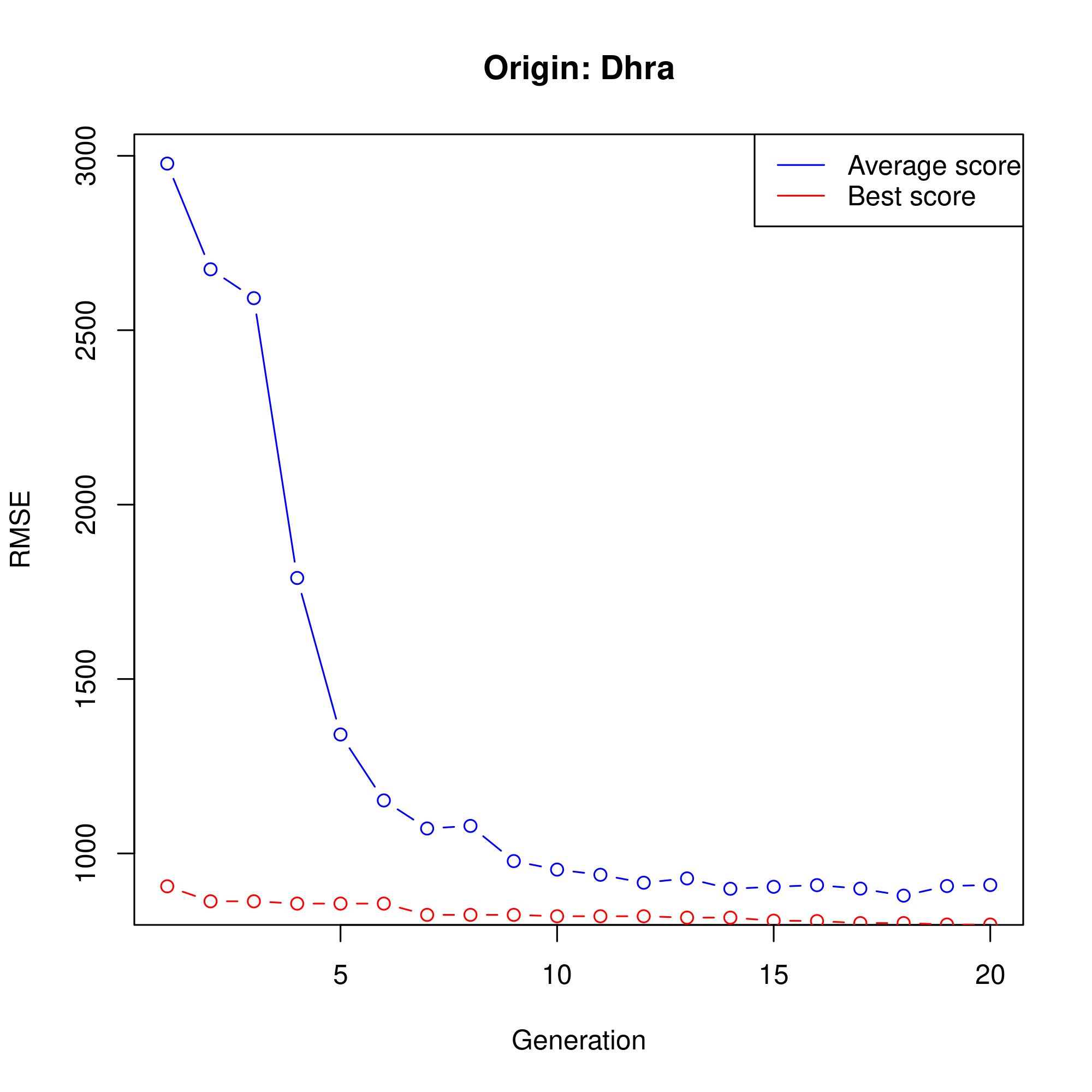

Supplement: S4 Fig — The average and the best (lowest) RMSE between simulated and radiocarbon dates are shown for each generation. (TIF) [file pone.0268482.s004.tif]
